# Associations between perceived overload and quality of care in dementia family caregivers in China: mediating role of familism and social support

**DOI:** 10.3389/fpubh.2024.1512778

**Published:** 2024-12-18

**Authors:** Ni Zou, Chan Cai, Xinyu Zhou, Shunian Chen, Jiabi Shi, Chongqing Shi

**Affiliations:** ^1^Institute of Nursing Research, Hubei Province Key Laboratory of Occupational Hazard Identification and Control, School of Medicine, Wuhan University of Science and Technology, Wuhan, Hubei, China; ^2^Department of Nursing, Xiangyang No.1 People’s Hospital, Hubei University of Medicine, Xiangyang, China

**Keywords:** perceived overload, familism, social support, quality of care, dementia, family caregivers

## Abstract

**Background:**

The quality of care (QoC) of people with dementia is an issue of widespread concern in public health. While perceived overload of family caregivers is thought to negatively affect QoC, the underlying mechanisms of this relationship are not well understood. This study aimed to examine the multiple mediating roles of familism and social support in the relationship between perceived overload and QoC among people with dementia (PwD) within the contemporary Chinese context.

**Methods:**

A cross-sectional study was conducted between February 2023 and October 2023 in three hospitals located in three cities in Hubei Province, China. A total of 213 PwD and their family caregivers were recruited. Participants completed a general demographic questionnaire, the Chinese version of the Overload Scale, the Social Support Rating Scale (SSRS), the Familism Scale (FS), and the Exemplary Care Scale (ECS). Data were analyzed using SPSS 26.0 and the PROCESS macro.

**Results:**

Perceived overload among family caregivers was directly related to QoC. Multiple mediation analysis revealed that the relationship between perceived overload and QoC was mediated by familism (effect: −0.111, 95% CI [−0.221, −0.034]) and social support (effect: −0.078, 95% CI [−0.163, −0.007]) both independently and serially (effect: −0.024, 95% CI [−0.054, −0.004]).

**Conclusion:**

Familism and social support serve as multiple mediators in the relationship between perceived overload and QoC. This underscores the importance of incorporating familism and social support into intervention strategies aimed at enhancing QoC.

## 1 Introduction

Dementia is a global issue, with the number of people with dementia (PwD) projected to reach 153 million by 2050 (1). In China, the prevalence of dementia is approximately 7%, affecting around 17 million people (2). More than 90% of PwD in China are cared for by informal family caregivers, influenced by traditional Chinese cultural values (e.g., Confucianism) and the limited availability of formal caregiving resources (3). The daily demands of caregiving, combined with the frustration caused by behavioral issues in PwD, place a significant burden on family caregivers, leading to severe perceived overload and potentially affecting the quality of care (QoC) provided to the patient (4). Low QoC has been associated with various adverse outcomes in PwD, such as higher mortality rates, a decline in life quality, frequent hospital readmissions, and an increased likelihood of institutionalization (5, 6). These consequences highlight the urgency of improving QoC for PwD to reduce these risks.

In China, family caregivers are often expected to take on caring roles due to traditional cultural values, particularly the cultural virtue of family care and filial piety in Confucianism. The strong sense of filial piety may partly explain why Chinese family caregivers of PwD experience more burdens and stresses compared to other racial/ethnic groups (7, 8). Meanwhile, with the rapid socio-economic development and changes in family structure in China, the traditional family care model is facing unprecedented challenges (9). As well as the underdeveloped Chinese dementia service system, family caregivers often lack the necessary support and resources (10). These further exacerbate their sense of overload. Therefore, it is becoming increasingly important to understand the mechanisms and conditions under which family caregivers’ perceived overload affects the QoC they provide to PwD in the Chinese context.

### 1.1 The influence of perceived overload on quality of care

QoC refers to the degree to which informal care satisfies the needs of the care recipient, both quantitatively and qualitatively, and it encompasses multiple dimensions (11). Christie et al. (12) identified three domains of QoC: (a) potential for harmful behavior (PHB), (b) adequacy of care, and (c) exemplary care (EC). EC involves a caregiver’s willingness and enthusiasm to provide care beyond fulfilling the basic needs of the older person, respecting their feelings, preferences, opinions, and values, while refraining from criticizing or reducing the individual’s limitations (13). This research focuses on EC because empirical evidence suggests that reciprocal and respectful caregiving may contribute more significantly to high-quality care than its quantity or adequacy (13). Previous research on the mechanisms affecting the QoC has largely focused on institutional settings such as nursing homes, with few studies directly examining the impact of stress and psychosocial factors on the informal QoC received by patients (14, 15). Perceived overload manifests as emotional exhaustion, stress, and fatigue when an individual is unable to align their needs or resources with the demands of a specific task or environment (16). To better understand its impact on QoC, the Stress Process Model (SPM) proposed by Pearlin et al. (17) provides a useful conceptual framework. This model suggests that perceived overload functions as a significant stressor, potentially affecting caregivers’ psychological state and behavioral attitudes, subsequently influencing the QoC they deliver in the context of family caregiving (17). There is substantial evidence indicating that perceived overload frequently results in poor QoC (18–21). For instance, Borghi et al. (20) have linked perceived overload with inappropriate emotional responses, a lack of patience, and reduced caregiving behaviors toward PwD during caregiving activities. Caregivers experiencing overload are prone to negative emotions such as elevated stress, exhaustion, and anxiety (22), which can impair their caregiving behaviors, potentially leading to neglect, impatience, or apathy in their interactions with care recipients (23). Building on these findings, we hypothesize that perceived overload negatively impacts QoC. While previous studies have explored the correlation between perceived overload and QoC, there is a gap in understanding the underlying mechanisms driving this relationship, which our study seeks to investigate.

### 1.2 The mediating role of familism

Familism is defined as an individual’s strong connection and commitment to their family members, which refers to values about support, interconnectedness, obligations and loyalty to the family (24, 25). Familism culture values are belonged to support resource in SPM, it is highly relevant to informal caregiving, as caregivers often take on the responsibility of caring for their relatives based on the principles of familism (26). Some studies have confirmed that familism is one of the factors affecting QoC and psychological health of family caregivers of individuals with dementia (27–29). In China, familism, one of the core social and cultural values, deeply rooted in Confucian principles, emphasizes family interconnection and support. Song et al. (30) found that family caregivers with a strong sense of familial obligation have greater expectations of receiving support from other family members at home. This family-centered value helps caregivers receive emotional and practical assistance when facing challenges, making their experience less negative and stressful, which is a key factor in providing high QoC (9, 31). According to Sociocultural Stress and Coping Model (SSCM), familism has been shown to help mitigate various stressors and enhance their ability to cope with stressful events, thereby promoting their psychological well-being and influencing the caregivers’ behavioral attitudes (25). Therefore, we hypothesize that perceived overload indirectly affects QoC through the mediation of familism. Although familism has gained increasing attention among Asian-American populations in North America, there is relatively little research on familism within the context of different ethnic groups in Asia (32). This study explores the impact of familism on informal caregiving within Chinese culture, aiming to provide new insights into the dynamics of family caregiving in the context of Chinese culture and potentially inform the development of support strategies and intervention measures.

### 1.3 The mediating role of social support

SPM has shown that social support is a key psychosocial factor affecting the relationship between the stressors experienced by family caregivers and their caregiving outcomes, as demonstrated by numerous empirical studies (33, 34). Social support refers to the various types of help and resources that individuals receive from their social networks, and it is a significant factor in shaping caregivers’ attitudes, behaviors, and psychological stress (35). In China, familism emphasizes interdependence among individuals, which increases the perceived social support. As a result, caregivers may be more inclined to seek and receive help (36), thereby reducing stress and improving their ability to provide QoC. Liang et al.’s research found that perceived overload can severely hinder a family caregiver’s ability to access and utilize social resources, which are a vital component of social support (37). This disruption impacts their capacity to receive practical assistance and emotional support from their social networks. Additionally, Bevan et al.’s study demonstrated that strong social support is closely associated with better QoC provided by family caregivers (38). Based on these findings, we hypothesize that social support mediates the relationship between perceived overload and QoC.

### 1.4 The chain mediation from familism to social support

Simultaneously, Family Ecosystem Theory (FET) (39) highlights the complex interactions between family members and their surrounding environment, showing how individual development is intricately shaped by these dynamics. Some researchers have argued that strong familism values increase individuals’ emotional reliance on family support networks and encourage pro-social behaviors in times of crisis or emergency (40, 41). As a result, familism may play a central role in shaping the social support available to family caregivers. Therefore, we propose that familism and social support act as sequential mediators between perceived overload and QoC.

### 1.5 The current research

The existing literature examines the relationships among the variables of perceived overload, familism, social support and QoC separately, but there remains a gap in research on the complex relationship between perceived overload and QoC among family caregivers of PwD within the unique cultural and social context of China. For the first time, we developed a chain mediation model to explore this relationship, using the SPM as the theoretical framework and introducing familism and social support as chain-mediated variables. We hypothesized that: (1) perceived overload negatively affects QoC; (2) familism mediates the relationship between perceived overload and QoC; (3) social support mediates the relationship between perceived overload and QoC; and (4) perceived overload influences QoC through the combined mediating effects of familism and social support. The findings aim to understand the complex interplay between cultural factors and social support within the context of caregiving, offering insights that could guide the development of interventions to improve caregiver well-being and the QoC for PwD.

## 2 Materials and methods

### 2.1 Study design, procedure, and participants

A cross-sectional research design was employed. Using a non-probability convenience sampling method, PwD attending three tertiary hospitals in three cities, Wuhan, Xiangyang, and Yichang, Hubei Province, China, between February 2023 and October 2023 were included in this study. Patients diagnosed with dementia according to the criteria established by the Diagnostic and Statistical Manual of Mental Disorders, 5th edition, aged 60 or above and receiving home care. Those who had received paid service care at home were excluded. All caregivers were the primary caregivers of PwD. Primary informal caregivers are those who meet the following conditions: (1) being adult informal caregivers, such as spouses, children, and other family members; (2) spending at least four hours per day on caregiving for no less than 3 months (42–44); (3) often accompanying patients to see a doctor, the best understanding of the patient’s condition, and basic living conditions; and (4) willing to participate in the study. Exclusion criteria were: (1) language and communication disorders, (2) severe physical or mental illness, (3) other major stressful events such as bereavement and divorce within past 3 months, or (4) planning to place the PwD in an older adult care facility within 6 months.

In this study, paper questionnaires were collected face-to-face and all investigators were uniformly trained. After being fully apprised of the purpose of the research, all participants granted written consent and filled out an anonymous questionnaire independently. If the respondents were illiterate, the answers were given in the form of questions and answers with the assistance of the investigators. The questionnaires were distributed and collected on the spot. After the questionnaire was completed, the investigators checked the questionnaire on the spot for omissions or obvious logical errors. If there were any problems, they were solved on the spot. Each questionnaire was completed within 20–30 min.

The sample size is based on a study that estimated the prevalence of dementia to be 7% (2). *u_α_* = 1.96, *δ* = 0.05, *p* = 7%. Based on the following formula (45), a minimum of 100 participants was required. The sample size was expanded by 20% to take into account factors such as sample loss or non-cooperation, ensuring a minimum of 125 participants. For structural equation modeling, a sample size exceeding 200 is recommended when dealing with more than 10 variables to ensure unbiased parameter estimates and convincing results (46). We finally collected a sample of 213 patients. The sample size met the requirements.


$$N=\frac{\mu_{\alpha }^{2}p\left(1-p\right)}{\delta^{2}}$$

### 2.2 Measures

#### 2.2.1 Demographic information

Utilizing a self-compiled questionnaire, we gathered demographic information for both the PwD and their caregivers. The information collected from PwD included age, gender, chronic disease, and activities of daily living (ADL). The ADL was assessed using the Barthel Index, which was completed by their caregivers. The data collected from the caregivers included age, gender, education level, place of residence, relationship with care recipients, living with care recipients, time of caring, length of care, self-rated health, affordability of living expenses.

#### 2.2.2 Perceived overload

Caregivers’ perceived overload was measured using the Overload scale (17). The 4-item Overload scale uses a 4-point Likert scale ranging from 1 (Not at all) to 4 (Completely), with higher scores implying higher levels of perceived overload. The Chinese version of the Overload scale was produced following Brislin’s guidelines (47), and the scale has proven highly reliable. The Cronbach’s *α* was 0.791 in this study.

#### 2.2.3 The quality of care (QoC)

Quality of care was assessed using Chinese version of the Exemplary Care Scale (ECS) revised by Lau et al. (48). The ECS contains 11 items and comprises two components: provide (items 1–5) and respect (items 6–11). Each item is rated on a 4-point scale (0 = never, 1 = sometimes, 2 = often, and 3 = always). The total score ranges from 0 to 33. A higher total score indicates a higher QoC provided by informal caregivers. The Exemplary Care Scale has shown good reliability and validity in Chinese populations (48). The Cronbach’s *α* was 0.816 in this study.

#### 2.2.4 Familism

The Familism Scale (FS) revised by Sabogal et al. (24) was used to assess familism. The scale has nine items, each rated on a five-point Likert scale ranging from 1 (‘strongly disagree’) to 5 (‘strongly agree’). It measures three distinct factors: familial obligations (items 1–2), familial support (items 3–5), and the family as a reference point (items 6–9). A higher score indicates a higher perception of familism. The Cronbach’s *α* was 0.734 in this study.

#### 2.2.5 Social support

The Familism Scale (FS) support was conducted using the Chinese version of the Social Support Rating Scale (SSRS) developed by Shuiyuan (49). The scale consists of 10-items which includes three dimensions: the subjective social support, objective social support, and the utilization of social support. Questions 1 to 4 and 8 to 10 are single-choice questions, with options (1), (2), (3), and (4) scoring 1, 2, 3, and 4 points, respectively. Question 5 asks about “the support and care received from family members,” rated on a 4-point scale from 1 (none) to 4 (full support). Questions 6 and 7 are assessed based on the number of options selected, which means number of sources of help. The total score for social support ranges from 1 to 66. The higher the score, the greater the level of social support. The Social Support Rating Scale has shown good reliability and validity in Chinese populations (50). The Cronbach’s *α* was 0.824 in this study.

### 2.3 Statistical analysis

SPSS version 26.0 and the PROCESS macro were employed to data analysis. *p* < 0.05 was considered to have statistical significance. Descriptive statistical analyses were conducted. Continuous variables were checked for normality with the P–P plots and characterized by means and standard deviations (SD). Categorical data were described frequencies and percentages. T-tests or one-way ANOVAs were calculated to compare the patients’ and caregivers’ demographic differences in the promotion of QoC. Multiple mediation models used these difference variables as covariates. The association among perceived overload, familism, social support and QoC were performed using Pearson’s correlation coefficients. Hypothesis 1 (perceived overload negatively impacts QoC), Hypothesis 2 (familism plays a mediating role between perceived overload and QoC), Hypothesis 3 (social support mediating the relationship between perceived overload and QoC) and Hypothesis 4 (familism and social support play a chain mediating role between perceived overload and QoC) were tested by performing the mediation test on the PROCESS macro program of SPSS 26.0 plug-in (51). Mediating effects were tested and validated using 5,000 Bootstrap resamples and bias-corrected 95% confidence intervals (CIs). A significant mediation effect exists if the lower confidence interval (LLCI) and upper confidence interval (ULCI) do not contain zero (52).

## 3 Results

### 3.1 Characteristics of care recipients and informal caregivers

Table 1 lists the demographic characteristics of care recipients and caregivers. 213 care recipients were aged 60 to 100 (77.29 ± 9.42) years. Most care recipients were male (54.9%, *n* = 117), with moderate to severe dependence (64.3%, *n* = 137). Among the 213 caregivers, the ages were ranged from 20 to 92, with a mean age of 59.06 (SD = 14.33) years, Most caregivers were female (64.8%, *n* = 138). Other socio-demographic descriptions were detailed in Table 1.

**Table 1:** One-way analysis of QoC of the study participants with different characteristics (*N* = 213).

Characteristic	Group	N (%)	Mean ± SD	*t/F*
Care recipients				
Age (years)	60–74	88 (41.3)	16.97 ± 4.60	−0.466
	≥ 75	125 (58.7)	17.28 ± 5.00	
Gender	Male	117 (54.9)	16.58 ± 4.76	−1.910
	Female	96 (45.1)	17.84 ± 4.85	
Chronic disease	No	22 (10.3)	15.68 ± 5.85	−1.510
	Yes	191 (89.7)	17.32 ± 4.69	
ADL	No dependence	20 (9.4)	21.15 ± 3.94	9.538^***^
	Mild dependence	56 (26.3)	18.43 ± 5.06	
	Moderate dependence	43 (20.2)	16.56 ± 4.41	
	Severe dependence	94 (44.1)	15.81 ± 4.44	
Caregivers				
Age (years)	18–44	30 (14.1)	17.43 ± 4.07	0.140
	45–59	78 (36.6)	17.18 ± 5.28	
	60–74	77 (36.2)	17.22 ± 4.28	
	≥ 75	28 (13.1)	16.57 ± 5.82	
Gender	Male	75 (35.2)	17.44 ± 5.02	0.644
	Female	138 (64.8)	16.99 ± 4.74	
Education level	Primary school or below	45 (21.1)	14.98 ± 5.07	9.920^***^
	Secondary school	94 (44.1)	16.87 ± 4.82	
	High school or above	74 (34.8)	18.82 ± 4.10	
Place of residence	Rural	70 (32.9)	14.96 ± 5.39	−4.467^***^
	Urban and town	143 (67.1)	18.22 ± 4.15	
Relationship with care recipients	Spouse	76 (35.7)	17.11 ± 4.91	5.453^**^
	Children	110 (51.6)	17.84 ± 4.63	
	Other family members	27 (12.7)	14.48 ± 4.62	
Living with care recipients	Yes	161 (75.6)	17.13 ± 4.89	−0.072
	No	52 (24.4)	17.19 ± 4.70	
Time of caring (h/d)	4 to <8	64 (30.1)	18.11 ± 4.66	3.265^**^
	8–12	42 (19.7)	18.45 ± 4.41	
	>12	107 (50.2)	16.05 ± 4.96	
Length of care (year)	<1	70 (32.9)	18.11 ± 4.26	6.271^**^
	1–5	99 (46.4)	17.52 ± 4.86	
	>5	44 (20.7)	15.02 ± 4.69	
Self-rated health	Poor	42 (19.7)	15.38 ± 4.31	5.814^**^
	Fair	98 (46.0)	16.94 ± 4.93	
	Good	73 (34.3)	18.45 ± 4.67	
Affordability of living expenses	Difficult	62 (29.1)	13.68 ± 4.54	28.476^***^
	Somewhat difficult	66 (31.0)	18.50 ± 4.29	
	Not difficult	85 (39.9)	18.64 ± 4.83	

*P < 0.05, **P < 0.01, ***P < 0.001.

### 3.2 Descriptive statistics and correlations among the main variables

The mean scores for perceived overload, familism, social support, and QoC are presented in Table 2. Additionally, Table 2 presents the results of the Pearson’s correlation analysis conducted on the study variables. QoC was negatively correlated with perceived overload (*r* = −0.621, *p* < *0.01*), and it was positively correlated with familism (*r* = 0.361, *p* < 0.01) and social support (*r* = 0.527, *p* < 0.01). Familism was negatively correlated with perceived overload (*r* = −0.305, *p* < 0.01), and positively correlated with social support (*r* = 0.272, *p* < 0.01). Social support was negatively correlated with perceived overload (*r* = −0.378, *p* < 0.01).

**Table 2:** Descriptive statistics and correlations among the variables (*n* = 213).

Variables	Mean ± SD	perceived overload	familism	social support	QoC
Perceived overload	9.63 ± 2.90	1			
Familism	33.38 ± 4.59	−0.305^**^	1		
Social support	39.25 ± 7.04	−0.378^**^	0.272^**^	1	
QoC	17.15 ± 4.83	−0.621^**^	0.361^**^	0.527^**^	1

^*^P < 0.05, ^**^P < 0.01, ^***^P < 0.001.

### 3.3 Multiple mediation model

**Figure 1:**
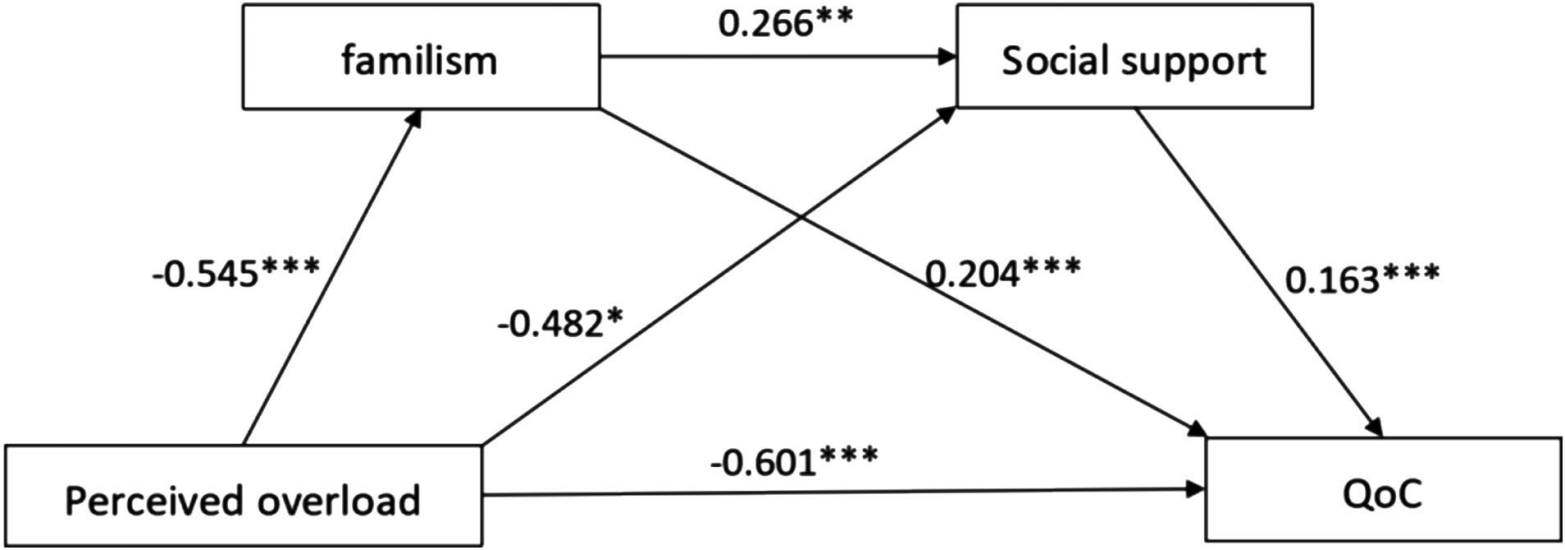
The multiple mediation model of familism and social support linking perceived overload and QoC of PwD (*N* = 213). QoC, quality of care. *p < 0.05, **p < 0.01, ***p < 0.001.

Significant covariates (i.e., the care recipient’s ADL, the caregiver’s level of education, place of residence, relationship with the care recipient, time of caring, length of care, self-rated health, and affordability of cost of living) in the univariate analyses were controlled for in the mediation model. Utilizing 5,000 bootstrapping samples, we investigated the relationship between perceived overload and QoC, taking into account the mediating roles of familism and social support. The results are summarized in Table 3. The total indirect impact of perceived overload on QoC was significant (indirect effect = −0.213, SE = 0.070, 95% CI [−0.363, −0.090]). Perceived overload indirectly affected QoC through familism (indirect effect = −0.111, SE =0.049, 95% CI [−0.221, −0.034]) and social support (indirect effect = −0.078, SE = 0.040, 95% CI [−0.163, −0.007]). Furthermore, perceived overload indirectly impacted QoC through familism and social support in serial (indirect effect = −0.024, SE = 0.013,95% CI [−0.054, −0.004]). The direct impact of perceived overload on QoC was significant (direct effect = −0.601, SE =0.107, 95% CI [−0.811, −0.391]) (see Figure 1).

**Table 3:** Summary results of the mediation analyses.

Path	Effect	SE	LLCI	ULCI
Total effect	−0.814	0.112	−1.035	−0.593
Direct effect	−0.601	0.107	−0.811	−0.391
Total indirect effect	−0.213	0.070	−0.363	−0.090
Indirect 1	−0.111	0.049	−0.221	−0.034
Indirect 2	−0.078	0.040	−0.163	−0.007
Indirect 3	−0.024	0.013	−0.054	−0.004

SE, standard error; LLCI and ULCI, lower level and upper level of the bias-corrected 95% bootstrap confidence interval. Direct effect = perceived overload → quality of care; Indirect 1 = perceived overload → familism → quality of care; Indirect 2 = perceived overload → social support →quality of care; Indirect 3 = perceived overload → familism → social support →quality of care.

## 4 Discussion

To the best of our knowledge, this is the first study to examine the role of familism and social support as mediators of the relationship between perceived overload and QoC among family caregivers of PwD in China. Different from Western caring culture, our research has revealed that both familism and social support are crucial resources in context of China. We combined SPM, SSCM and the FET to gain a better understanding of cultural influences in the care of PwD at home in China. Theoretical models developed based on western culture emphasize the role of social support, but familism of culture value is also important in the Chinese context.

Our study found that perceived overload was negatively associated with QoC, which aligns with previous research (19), confirming Hypothesis 1. In general, findings indicate that the QoC received by patients is significantly affected by various stressors experienced by family caregivers during daily caregiving activities. This highlights the key role of perceived overload in maintaining QoC. Specifically, caregivers experience perceived overload when the demands of care exceed their ability to cope, often resulting in fatigue and burnout (53). Hence, their capacity to provide care and support is reduced, leading to a decline in the QoC for PwD (54).

Our findings indicated that familism moderated the relationship between perceived overload and QoC, supporting Hypothesis 2. Consistent with previous studies, the attitudes and behaviors of family caregivers toward patients are influenced by both stressors and family environmental factors (39, 55). Family caregivers may experience fatigue and burnout due to intense perceived overload, which reduces their psychological resilience and coping abilities, making it difficult for them to fulfill family responsibilities (56). From the perspective of the “family obligation” dimension of familism, caregiving is seen as a family duty. Xu (57) research shows that obligation such as filial piety, helping family members, and maintaining family honor remains highly valued in contemporary Chinese families. Traditional Chinese culture emphasizes love and filial piety, this obligation extends beyond material support to include emotional companionship and respect (31). In addition, the family support is another dimension of familism. It refers to the emotional and practical assistance that family members provide to each other, which greatly enhances the psychological resilience of caregivers, allowing caregivers to better cope with stress and challenges (24). Furthermore, family as a reference dimension is the third dimension of familism, which highlights the pivotal role of family in shaping individual decision-making and behavior. Caregivers are likely to strive to provide higher QoC in line with their family’s expectations and values (9, 24). As a result, caregivers with strong familism are more likely to provide high QoC in a compassionate and patient manner during caregiving activities. Therefore, understanding the interactions among perceived overload, familism and QoC is crucial for providing culturally grounded and effective support, ultimately promoting an environment for high-quality care.

The results also suggested that the mediating role of social support in perceived overload and QoC, supporting Hypothesis 3. High levels of social support have been shown to buffer the effects of perceived overload on family caregivers, aligning with the SPM (17). Family caregivers perceive higher levels of social support may reduce the reliance on negative coping strategies and, in turn, positively impacts the QoC provided by caregivers (58). Furthermore, when family caregivers receive help and care from family or friends, their fatigue is significantly reduced, and they are more willing to offer proactive care (59). In this study, the population from urban areas accounts for more than half. In China, there is a significant disparity in the level of social support between urban and rural areas, primarily due to differences in economic development levels, distribution of social resources, and infrastructure construction (10). According to data from the WHO in 2021, formal agreements and joint plans for dementia care in China are not yet fully developed, and the availability of social and economic protections is insufficient (60). Especially in rural areas, fewer services, accessing and utilizing limited resources make it harder for caregivers to obtain support (61).

The results also indicated that perceived overload influences QoC among caregivers through the serial mediation effects of familism and social support, confirming Hypothesis 4. The study suggests that familism may play a central role in shaping the social support available to family caregivers. Familism emphasizes emotional closeness and mutual assistance among family members (24). Emotional support allows family caregivers to feel cared for and understood by their family members when facing stress and challenges, thereby increasing their psychological resilience and sense of social support (62). By encouraging family members to help one another during difficult times, family caregivers can receive practical assistance (e.g., patient care, household chores, financial support) from other family members. As a result, family caregivers with high familism may receive more family support due to the close-knit nature of family relationships. In China, familism is deeply connected to cultural heritage and the reinforcement of traditional values. Mutual help and support among family members reflect the principles of filial piety and family harmony central to traditional Chinese culture, and these cultural values are passed down and strengthened through familism (31). When caregivers perceive overload, they rely not only on internal cultural values but also on external social support networks to cope. This dual support, cultural and social, helps reduce the physical and psychological stress experienced by family caregivers during the caregiving process, making them more attuned to the patient’s needs and feelings and more likely to provide exemplary care.

### 4.1 Implications

This study provides a reference for interventions of improving QoC of PwD. By establishing correct cultural values and maintaining good family relationships, caregivers may experience greater family cohesion, a stronger sense of responsibility, and deeper emotional bonds, leading to more patient and compassionate caregiving (10). Social support interventions, such as support groups and skill-building workshops, could provide higher-quality care (63). Providing caregivers with necessary assistant social support resources is crucial to improve QoC (64). This suggests that the government should integrate various available resources, establish the caregivers of PwD social support system that is suitable for China’s national conditions.

### 4.2 Limitations

Despite these strengths, this study has some limitations. Firstly, the cross-sectional design limits causal inferences. Future research should consider a longitudinal approach to better capture the dynamic effects of perceived overload, familism, and social support on QoC over time. Secondly, self-reported data on perceived overload, familism, social support, and QoC was used. Reliance on self-reported data may introduce bias due to participants’ subjective interpretations and their tendency to respond in a socially desirable manner. Future studies could enhance objectivity by incorporating additional assessment methods like observations or data obtained from qualitative interviews. Thirdly, conducted in three hospitals in central China, our findings may not be generalizable to all dementia caregivers in China, especially given the country’s regional and economic diversity. Future research with larger and more diverse samples should test the consistency of results across different regions and cultural settings. Lastly, while focusing on familism and social support, our study did not include other potential factors such as family financial resources, caregiver mental health, and availability of external support institutions. Future research could benefit from a broader range of variables for a more comprehensive evaluation of QoC.

## 5 Conclusion

This study clarified the mediating pathway between familism and social support among Chinese family caregivers of PwD. Familism and social support act as mediators in the relationship between perceived overload and QoC. By targeting interventions to reduce perceived overload while simultaneously enhancing the beneficial effects of familism and social support, we may achieve a direct enhancement in QoC. Additionally, alleviating perceived overload in caregivers of PwD may lead to an indirect improvement in QoC.

## Data Availability

The raw data supporting the conclusions of this article will be made available by the authors, without undue reservation.
